# SNAP II and SNAPPE II as Predictors of Neonatal Mortality in a Pediatric Intensive Care Unit: Does Postnatal Age Play a Role?

**DOI:** 10.1155/2014/298198

**Published:** 2014-02-26

**Authors:** Mirta Noemi Mesquita Ramirez, Laura Evangelina Godoy, Elizabeth Alvarez Barrientos

**Affiliations:** Hospital General Pediátrico “Niños de Acosta Ñú”, Avenida de la Victoria and Bacigalupo, Reducto, 2160 San Lorenzo, Paraguay

## Abstract

*Introduction*. In developing countries, a lack of decentralization of perinatal care leads to many high-risk births occurring in facilities that do not have NICU, leading to admission to a PICU. *Objective*. To assess SNAP II and SNAPPE II as predictors of neonatal death in the PICU. *Methodology*. A prospective study of newborns divided into 3 groups according to postnatal age: Group 1 (G1), of 0 to 6 days; Group 2 (G2) of 7 to 14 days; and Group 3 (G3), of 15 to 28 days. Variables analyzed were SNAP II, SNAPPE II, perinatal data, and known risk factors for death. The Hosmer-Lemeshow test and the receiver operating characteristics (ROC) curve were used with SPSS 17.0 for statistical analysis. An Alpha error <5% was considered significant. *Results*. We analyzed 290 newborns, including 192 from G1, 41 from G2, and 57 from G3. Mortality was similar in all 3 groups. Median SNAP II was higher in newborns that died in all 3 groups (*P* < 0.05). The area under the ROC curve for SNAP II for G1 was 0.78 (CI 95% 0.70–0.86), for G2 0.66 (CI 95% 0.37–0.94), and for G3 0.74 (CI 95% 0.53–0.93). The area under the ROC curve for SNAPPE II for G1 was 0.76 (CI 95% 0.67–0.85), for G2 0.60 (CI 95% 0.30–0.90), and for G3 0.74 (CI 95% 0.52–0.95). *Conclusions*. SNAP II and SNAPPE II showed moderate discrimination in predicting mortality. The results are not strong enough to establish the correlation between the score and the risk of mortality.

## 1. Introduction

Birth weight has classically been considered as the most significant predictor of neonatal mortality. In developed countries, improvement of neonatal care, advances in neonatal ventilation, and in particular the use of pulmonary surfactant have not only reduced preterm neonatal mortality, but also increased survival for extremely premature infants. Other factors have been found to affect morbidity and mortality, among them the severity of disease upon hospitalization [1–3].

In the 1990s, Richardson et al. developed a system of assessment for the most important physiological variables affecting mortality in the first hours following admission. Each variable was assigned points based on the values found, and the result was the Score for Neonatal Acute Physiology (SNAP) [4].

SNAP assesses the worst clinical status found in the first 24 hours after admission using points assigned to 26 physiological variables: the higher the score, the greater the risk of death. With the Score for Neonatal Acute Physiology Perinatal Extension (SNAPPE), 3 additional variables were added: birth weight, the Apgar score, and being small for gestational age [4]. Due to the time needed to complete scoring, the authors subsequently developed a simplified version of the score, using only 5 variables to be measured within 12 hours of admission. The simplified scoring system was designated SNAP II and its perinatal extension SNAPPE II. These scoring systems have been validated in studies with large numbers of patients and have been shown to be good predictors of mortality in newborns in neonatal intensive care units (NICU). Use of the scoring systems has also allowed comparison of mortality rates from NICUs of different perinatal hospitals adjusted by severity of the disease at admission [5].

The clinical and epidemiological characteristics of newborns admitted to intensive therapy units in specialized hospitals present different clinical and epidemiological characteristics: they frequently of greater birth weight and are subjected to transfer procedures and show generally higher mortality [6]. Among risk factors cited for mortality of newborns managed in the NICU of pediatric hospitals are the transfer from other NICUs, presence of congenital malformations, and a requirement for surgery [6, 7]. It could be said that newborns entering polyvalent pediatric intensive care units (PICU) constitute a special subgroup of newborns. In developing countries, a lack of decentralization of perinatal care leads to many high-risk births occurring in facilities that do not have NICU, meaning that sick newborns must be transferred to specialized hospitals that may not possess an NICU, or may be overloaded with patients, leading to admission to a PICU. A group of newborns also exist who present with disease between the third and fourth weeks of life and require neonatal intensive care. Transfer of these newborns from one hospital to another is frequently done by means that are not adequate.

Our prospective study was done with the object of assessing whether SNAP II and SNAPPE II can predict mortality in this newborn population.

## 2. Material and Methods

We performed a prospective, observational, cohort study to assess SNAP II and SNAPPE II in a newborn population admitted to the PICU of the *Niños de Acosta Ñu* general pediatric hospital in Asunción, Paraguay. We included newborns with gestational ages between 28 and 42 weeks admitted to the PICU between January 2010 and December 2011. The newborns were divided into 3 groups according to postnatal age at admission: Group 1 (G1) was newborns with postnatal ages of from a few hours to 6 days, Group 2 (G2) were between 7 and 14 days of age, and Group 3 (G3) was from 15 to 28 days of age. Division of the population into 3 postnatal age groups at admission was decided based on the particular characteristics of each age group. Group 1 was comprised of the youngest newborns, who presented predominantly respiratory disease and symptoms of perinatal asphyxia. Group 2 was generally more stable newborns who had been hospitalized in less-well equipped hospitals and were transferred to the pediatric hospital by the reference counterreference system (including exchange of less seriously ill patients to less specialized institutions to avoid overloading), or due to complications and a requirement for mechanically assisted ventilation. Group 3 was comprised of newborns of more than 2 weeks of life who were admitted largely due to symptoms of severe bronchiolitis or late-onset neonatal sepsis.

The transfer of newborns from rural areas of the country to hospitals in the city is not always performed under appropriate conditions, for example, ambulance equipped with transport incubator and trained health workers. This is due to the small number of ambulances with proper equipment and lack of trained personnel. Many of the infants hospitalized in the pediatric hospital “Children of Acosta Nu” come from rural areas.

Variables for SNAP II and SNAPPE II scoring were taken from the patient medical records on a form created for this purpose within 12 hours of hospitalization. We excluded newborns who died within 12 hours of admission and those with congenital malformations incompatible with life. To determine the SNAPPE II score, newborns were excluded who did not receive immediate care at a health care institution (home childbirth), due to their lack of birth weight figures and Apgar tests.

Variables other than the scores analyzed were birth weight, gestational age, sex, Apgar test at 1 min. and 5 min., place and type of parturition, postnatal age at admission, intrauterine growth restriction, transfer from other hospitals, and congenital malformations. The clinical progress of patients was analyzed according to surgical intervention, entry to mechanically assisted ventilation (MV), and hospital discharge (living or dead).

For data analysis, the contingency table, Chi Square test, proportions, comparison of medians, and parametric and nonparametric means were used according to type, distribution, and variance of the variables. Analyses of true and false positives for each scoring value were done by calculating the area under the curve (AUC) using the receiving operating characteristic curve (ROC) and Hosmer-Lemeshow goodness-of-fit test for calibration of scoring using SPSS 17.0. An Alpha error of less than 5% was considered significant.

*Ethical Considerations*. Confidentiality of data was maintained at all times. Patient identifiers, for example, names, addresses, and so forth, were removed after data acquisition and subjects were then identified by study numbers. The protocol was approved by the hospital research and ethics committee (approval number 0022).

## 3. Results

In the period from January 2010 to December 2011, 350 newborns were admitted to the polyvalent PICU of our hospital, of which 60 were excluded: 2 due to congenital malformations incompatible with life, 3 due to death prior to 12 hours after admission, and 55 due to the score having not been provided prospectively. We analyzed 290 newborns. Of the 290, 192 (66%) were assigned to Group 1 (G1), 41 (14%) to Group 2 (G2), and 57 (20%) to Group 3 (G3).

No difference was found in perinatal data between the 3 groups in birth weight or percentage of low birth weight (LBW), very low birth weight (VLBW), gestational age, Apgar score at 1 min. and at 5 min., sex, place and type of parturition, or presence of intrauterine growth restriction (IUGR). Apgar score and birth weight were obtained for 232 newborns (data for 58 newborns, 39 from G1, 5 from G2, and 14 from G3 were unavailable due to home births or inability to verify data with the perinatal birth record) (Table 1).

Differences were found between groups for known risk factors in our population, including transfer from other hospitals (prior hospitalization) in the G2 group of 30 of 41 (73%); in the G1 group of 109 of 192 (57%:); and the G3 group of 19 of 57 (33%) (*P* < 0.01). The largest percentage of perinatal asphyxia was found in the G1 group, with 20% (39/192), compared to 10% (9/41) in the G2 group, and 2% (1/57) in the G3 group (*P* < 0.05). No differences were found in other risk factors analyzed (Table 2).

Overall mortality was 71 of 290 (24%). Mortality by age group was G1 52 of 192 (27%); G2 7 of 41 (17%); and G3 12 of 57 (21%). Although Group G1 had the highest mortality rate, the difference compared to other groups was not significant (OR 1.45 [CI 95% 0.8–2.2] *P* > 0.05).

Analysis of severity at admission measured by SNAP II (*n* = 290) scores showed higher values for newborns who died compared to those discharged alive in all 3 groups. Median SNAPPE II scoring (taken in 232 newborns) was also higher for newborns who died compared to those who survived in Groups 1 and 3, but not in Group 2, for which analysis did not show statistical significance (Table 3).

**Figure 1:**
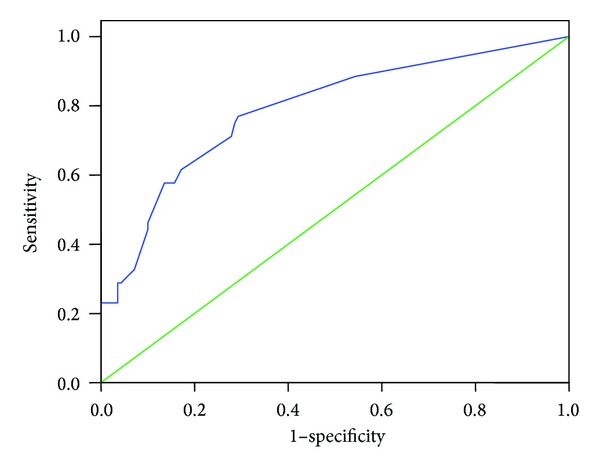
Analysis using the ROC curve showed that the area under the curve using SNAP II scores for Group 1 was 0.78 (CI 95% 0.71– 0.86).

Analysis using the ROC curve showed that the area under the curve using SNAP II scores for G1 was 0.78 (CI 95% 0.71–0.86) Figure 1. While for SNAPPE II (*n* = 153) it was 0.75 (CI 95% 0.67–0.84). The Hosmer-Lemeshow goodness-of-fit test result was 0.7.

For G2, the SNAP II score was 0.66 (CI 95% 0.37–0.94), while for SNAPPE II (*n* = 36) it was 0.60 (CI 95% 0.30–0.90). For G3, the SNAP II score was 0.74 (0.53–0.93), while for SNAPPE II (*n* = 43) it was 0.74 (0.52–0.95).

## 4. Discussion

No significant differences in severity scores were found between the 3 groups of newborns of different postnatal ages. The median SNAP II score was significantly higher in newborns who died compared to those who survived in all 3 groups. The SNAPPE II score was also higher in newborns who died from Groups 1 and 3, but not in Group 2, which we attribute to the small number of patients. Analysis of the ROC curve for both SNAP II and SNAPPE II showed an area under the curve with moderate values in Groups 1 and 3, but not for Group 2, as due to the small number of patients analyzed, with 7 deaths, a good curve could not be generated. These results are similar to those found by the authors of a study carried out at the same polyvalent pediatric intensive care unit of the hospital from 2006 to 2009, and in which the SNAP II and SNAPPE II scores were determined retrospectively in a group of 288 newborns with characteristics similar to those of our patients but analyzed as a single group without considering postnatal age. We found that both scores in that study showed higher values for newborns who died compared to survivors, with analysis of the ROC curve showing an area under the curve for SNAP II of 0.79 (CI 95% 0.72–0.85) and for SNAPPE II of 0.77 (CI 95% 0.69–0.86) [8]. Those findings were similar to those of the newborns in Group 1 of our study.

We have not found studies validating SNAP II and SNAPPE II scoring in populations of newborns with characteristics similar to our sample. Vasudenan et al. carried out a study using SNAP scoring in India in a population of newborns with an average postnatal age of 13 days who had been admitted to a polyvalent PICU similar to our own. In the 97 newborns analyzed scoring was significantly higher in patients who died compared to survivors and the ROC curve showed an area under the curve of 0.77 (CI 95% 0.68–0.87) [9]. These results are comparable to ours despite the use of a more complex scoring system with a larger number of physiological variables and greater time required for completion.

In another study, with a population comparable to ours in terms of postnatal age and being carried out in a developing country, although in a neonatal intensive care unit (NICU) and using different analyses, a high SNAP II score and low Apgar at 5 min. were associated with neonatal mortality in regression analysis [10].

Use of SNAP II and SNAPPE II in newborns with postnatal ages greater than 24 hours has been assessed in various studies and in specific situations in neonatal units, with varied results. In some, they did not predict mortality, sepsis, or enterocolitis [11]. In others, such as that carried out at a NICU in India, SNAP II scoring was analyzed as a predictor of mortality in very low birth weight (VLBW) newborns within average postnatal age of 4 days and diagnosis of severe septicemia. SNAP II scoring was done within 12 hours of onset of signs and symptoms. Patients who died showed a significantly higher score than those who survived. The cutoff point was 40, with a positive predictive value of 88% [12]. A similar cutoff point was used by Nakwan et al., who assessed SNAP II in patients with persistent pulmonary hypertension. Although it showed moderate discrimination in the study population (0.71 [CI 95% 0.56–0.88]), patients with a score ≥43 showed higher risk of death [7]. In our study the very low birth weight (VLBW) population was not analyzed as a group due to the small number of such patients.

The high percentage of congenital malformations observed in our study population is explained by our hospital being a neonatal surgery hospital of reference. It has also been a cardiovascular surgery hospital of reference for the last two years, leading to increased admission of newborns with congenital cardiopathies, who however were not included in our study as they are managed by the intensive care unit of the pediatric cardiology department. Published reports exist of validation of SNAP and SNAP II in newborns with congenital cardiopathies and other malformations such as congenital diaphragmatic hernia, for which they were not very good predictors of mortality [13, 14].

As a specialized pediatric hospital, 100% of newborns admitted are transported, whether from their homes or other hospitals. One very important variable is neonatal transport, which can influence clinical deterioration of the patient at admission [15]. In the population we studied, transport of the majority of patients is not done appropriately or with prior referral, meaning that the transport risk index of physiologic stability (TRIPS) cannot be done due to a lack of pretransport data.

In our study, SNAP II and SNAPPE II scoring showed better discrimination as predictors of mortality in the group of newborns of lowest postnatal age at admission (Group 1), but this was much lower than that reported in newborns in perinatal hospitals. This group of newborns comprised the group with the largest number of patients, thereby permitting better analysis. The newborns in this group had higher severity scores at admission compared to those from Groups 2 and 3, and mortality among them was also higher, although not reaching significance.

It is possible that mortality in the newborn population we studied is associated with other factors aside from severity at admission, such as neonatal transport and nosocomial infections.

## Figures and Tables

**Table 1:** Perinatal data of the three groups *n* = 290. LBW (low birth weight); VLBW (very low birth weight) IUGR (intrauterine growth restriction).

	Group 1	Group 2	Group 3	*P*
Birth weight (g) Medians (range) (*n* = 232)	2900 (670–5100)	3000 (1050–4710)	2900 (1070–4500)	NS
LBW (%)	36	42	27	NS
VLBW (%)	12	15	5	NS
Gestational age Medians (range) (*n* = 232)	37 (27–42)	37 (27–40)	38 (28–40)	NS
Apgar score 1 min	6 (1 – 9)	7 (2–9)	7 (2–9)	NS
Apgar score 5 min Medians (range) (*n* = 232)	9 (3–10)	8 (2–9)	9 (5–9)	NS
Male sex (%)	64	54	51	NS
Delivery (%)				
Vaginal	66	73	79	NS
Caesarean	34	27	21	NS
Hospital birth (%) (*n* = 232)	80	88	68	NS
Home birth (*n* = 58)	20	12	22	NS
IUGR (%)	27	24	28	NS

**Table 2:** Mortality risk factors in each group studied. MV (mechanical ventilation).

	Group 1	Group 2	Group 3	*P*
SNAP II (*n* = 290) (median)	10	5	6	<0.05
SNAPPE II (*n* = 232) (median)	13	7	8	<0.05
Prior hospitalization (%)	57	73	33	<0.05
Congenital malformations (%)	30	24	26	NS
Surgery (%)	29	19,5	21	NS
Perinatal asphyxia (%)	20	10	2	<0.05
Nosocomial infection (%)	33	19,5	21	NS
MV (%)	60	37	40	NS

**Table 3:** Median of SNAP II and SNAPPE II score of the three groups and the condition at discharge.

Group	Condition at discharged	SNAP II	*P*	SNAPPE II	*P*
G1	Alive	5	<0.05	5	<0.05
	Deceased	16		22	
G2	Alive	0	<0.05	0	>0.05
	Deceased	10		8	
G3	Alive	0	<0.05	0	<0.05
	Deceased	13		17	
